# Microbial therapeutics for canine periodontal disease: current status and future perspectives

**DOI:** 10.3389/fvets.2025.1748968

**Published:** 2026-01-12

**Authors:** Seok Bin Yang, Ji-Hoi Moon

**Affiliations:** 1 Department of Oral Microbiology, College of Dentistry, Kyung Hee University, Seoul, Republic of Korea; 2 Department of Dentistry, Graduate School, Kyung Hee University, Seoul, Republic of Korea

**Keywords:** canine periodontal disease, microbial therapeutics, oral microbiome, probiotic and postbiotic therapy, synthetic biology

## Abstract

Periodontal disease in dogs arises from ecological disruption of the oral microbiome. Sequencing-based studies and quantitative molecular analyses reveal characteristic dysbiotic transitions in affected dogs, with coordinated increases in *Porphyromonas gulae*, *Prevotella*, and *Treponema*, along with *Treponema denticola* and *Tannerella forsythia*, together with a loss of commensal-dominated genera such as *Moraxella*, *Capnocytophaga*, and members of the *Neisseriaceae* family. Rather than being driven by a single dominant pathogen, accumulating evidence indicates that canine periodontitis is driven by polymicrobial synergy within dysbiotic biofilms. This ecological perspective has stimulated growing interest in microbial therapeutics aimed at modulating community structure and function instead of relying solely on broad-spectrum antibiotics. Probiotics and postbiotics show potential in reducing halitosis and modulating epithelial innate immune responses. Bacteriophage-based approaches and predatory bacteria exhibit strain-specific antimicrobial activity in preclinical human or *in vitro* models, although their relevance to canine oral disease remains unvalidated. Synthetic biology and CRISPR-based antimicrobial systems provide conceptual frameworks for genotype-targeted modulation of virulence. Remaining challenges include transient microbial persistence, limited veterinary clinical evidence, biosafety concerns, and the absence of standardized regulatory pathways. Collectively, emerging microbial therapeutics highlight the potential but also the current limitations of ecology-guided, non-antibiotic strategies for canine periodontal therapy.

## 1 Introduction

Periodontal disease is one of the most common oral conditions in dogs. Its prevalence increases with age and affects a substantial proportion of adult animals. Clinical and epidemiological studies further indicate that disease prevalence increases with age but decreases with body weight, with toy and small breeds exhibiting the highest susceptibility (1, 2). It is characterized by chronic inflammation and progressive destruction of periodontal tissues, resulting from complex interactions between the host immune response and the resident oral microbiota (3). Recent advances in microbial sequencing have shifted the understanding of canine periodontal disease from an infection-based model to one centered on microbial dysbiosis, defined as a disruption of the symbiotic oral microbial ecosystem associated with functional and compositional imbalance leading to pathogenic biofilm development and inflammatory amplification (4–7). In human periodontology, similar conceptual transitions from single-pathogen infection models to dysbiosis-driven, community-level frameworks have been synthesized in recent reviews (7–9), providing a useful theoretical background for interpreting emerging canine data.

Despite improved awareness of its microbial etiology, current veterinary management remains dominated by mechanical scaling and broad-spectrum antibiotics. Mechanical plaque removal by professional debridement remains the cornerstone of periodontal therapy, while antimicrobial agents are frequently used as adjuncts. However, these interventions provide only temporary relief, often disrupting beneficial commensal communities and contributing to development of antimicrobial resistance (3). In routine clinical practice, daily homecare, particularly tooth brushing, is also widely recommended as a primary preventive strategy to delay plaque accumulation and disease progression. Consequently, attention has turned toward ecological modulation, defined as therapeutic strategies aimed at restoring a balanced oral microbiome rather than indiscriminately reducing bacterial load.

Microbial-based therapeutics—including probiotics, postbiotics, bacteriophages, and engineered microbial systems—have emerged as promising alternatives. Probiotics and prebiotics are now widely recognized as tools for reshaping dysbiotic oral biofilms through competitive exclusion, metabolic modulation, and host immune regulation (10, 11). Early studies in dogs have demonstrated that administration of beneficial microbes or their metabolites can reduce halitosis-associated bacteria and improve epithelial defense responses (12–15). In parallel, work in oral microbial ecology indicates that periodontal disease progression reflects polymicrobial synergy and community-level functional shifts within dysbiotic biofilms rather than the action of a single dominant pathogen (7–9, 16), emphasizing the need for precision modulation rather than broad suppression of microbial communities. Recent quantitative and screening-based canine studies have further demonstrated that multiple periodontal pathogens, including *Porphyromonas gulae*, *Treponema denticola*, and *Tannerella forsythia*, increase in parallel with disease severity (17, 18), supporting a multi-species ecological model of canine periodontitis.

However, no existing review has comprehensively integrated current knowledge of canine oral dysbiosis with the rapidly advancing field of microbial therapeutics, particularly emerging approaches that target pathogen virulence at the genotype or functional level. Moreover, most available summaries focus either on individual microbial taxa or on conventional antimicrobial strategies, leaving a critical gap in the synthesis of ecology-guided, non-antibiotic microbial interventions for canine periodontal disease. The present mini review therefore synthesizes recent developments in understanding the canine oral microbiome and evaluates the potential probiotics, postbiotics, bacteriophages, and synthetic-biology–engineered microbes as precision tools for ecological restoration and periodontal disease management in dogs.

## 2 Current understanding of the oral microbiome in dogs

The canine oral microbiome represents a complex ecological network composed of commensal, opportunistic, and pathogenic microorganisms. In healthy dogs, the oral bacterial community is dominated by aerobic and facultative taxa such as *Moraxella*, *Capnocytophaga*, and members of the *Neisseriaceae* family, which are associated with colonization resistance, epithelial integrity, and immune homeostasis (4, 5). These commensals participate in nutrient cross-feeding and signaling pathways that maintain ecological stability. Most current insights into these communities derive from 16S rRNA gene sequencing and targeted quantitative PCR approaches, which provide high-resolution taxonomic profiles but limited functional and strain-level resolution (4, 5, 17). Direct functional activity of canine oral biofilms has rarely been quantified, although metatranscriptomic approaches in human oral microbiome research have demonstrated that transcript-level analyses can reveal active metabolic pathways and virulence expression not predictable from taxonomic composition alone (19).

**Figure 1:**
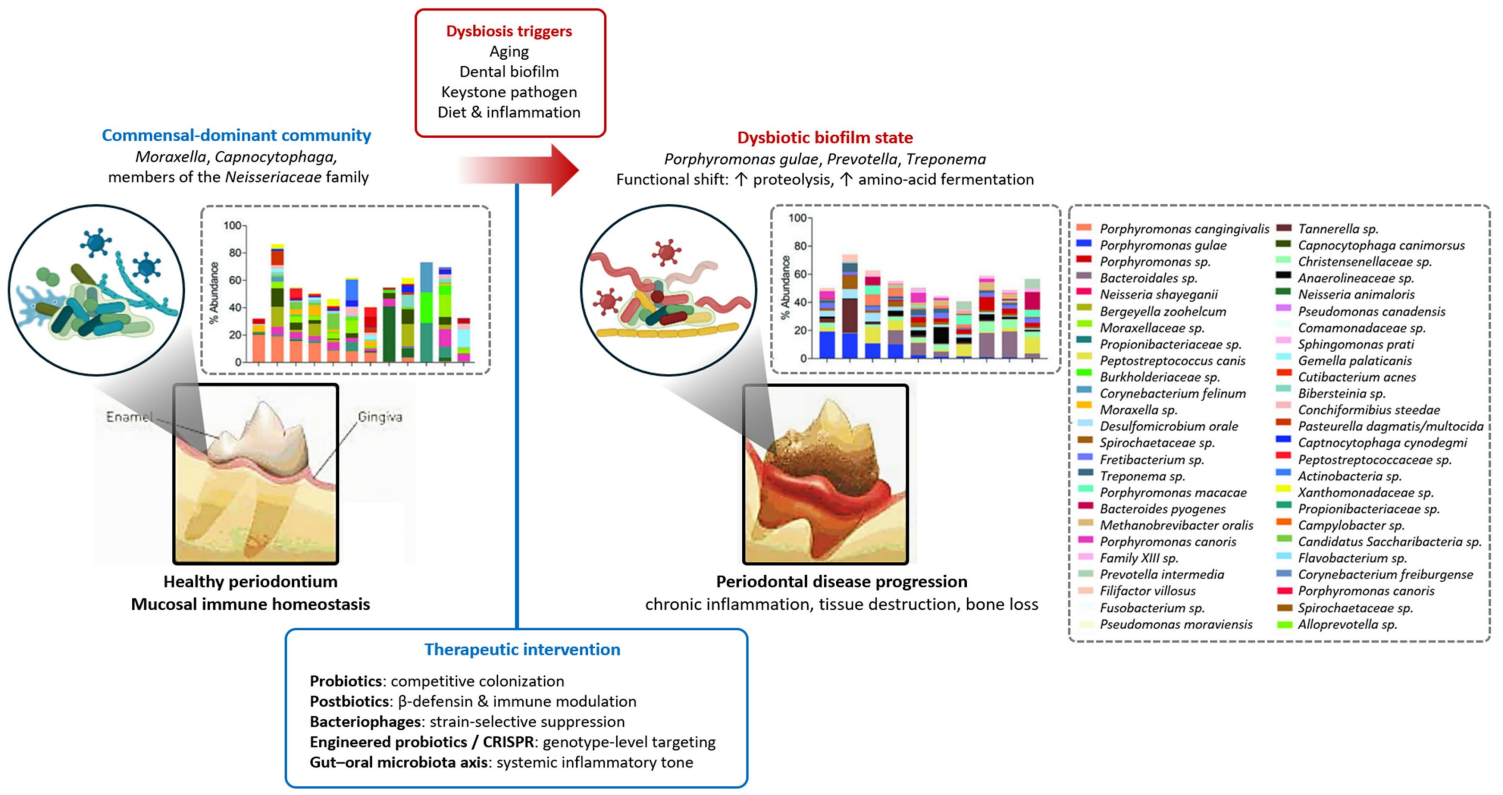
Conceptual overview of oral microbiome dysbiosis and microbial therapeutic strategies in companion animals. Healthy oral communities dominated by *Moraxella*, *Capnocytophaga*, and members of the *Neisseriaceae* family transition toward dysbiotic biofilms enriched with *Porphyromonas gulae*, *Prevotella*, and *Treponema* under the influence of aging, biofilm accumulation, and inflammation. Microbiome composition plots are adapted from Niemiec et al. (4), illustrating compositional and functional shifts toward enhanced proteolytic metabolism (5, 20). The lower panel summarizes microbial therapeutics conceptually targeting these transitions: probiotics, postbiotics, bacteriophages, engineered probiotics/CRISPR antimicrobials, and gut–oral microbiota modulation.

During periodontal disease, this ecological equilibrium is disrupted, leading to enrichment of anaerobic and proteolytic organisms such as *Porphyromonas gulae*, *Prevotella*, *Treponema*, and to a lesser extent *Fusobacterium* (5). Recent quantitative PCR–based analyses in large canine cohorts further demonstrate parallel increases in multiple periodontal pathogens, including *P. gulae*, *Treponema denticola*, and *Tannerella forsythia*, in association with disease severity (17, 18). Functional inference analyses suggest that this compositional shift is accompanied by enhanced proteolysis, amino acid fermentation, and inflammatory metabolic potential within dysbiotic biofilms (5). These “functional inference” approaches, typically based on predictive metagenomic algorithms, estimate potential metabolic activity from taxonomic profiles rather than directly measuring gene expression or metabolite fluxes. Complementary age-stratified analyses further show that similar increases in proteolytic and fermentation-associated pathways occur with aging, suggesting that functional remodeling of the oral microbiome may precede or predispose to periodontal dysbiosis (20). Regarding *Fusobacterium*, recent reports indicate that its relative contribution to canine periodontal dysbiosis may vary depending on disease stage and sampling niche, warranting cautious interpretation of its pathogenic role across studies (2, 21, 22). Together, these findings highlight that canine periodontal disease reflects not merely an overgrowth of pathogenic taxa, but a broader ecological and metabolic reorganization of the oral microbiome (Figure 1).

Importantly, the salivary microbiota differs substantially from supragingival and subgingival biofilm microbiota in dogs. Niche-resolved studies demonstrate that bacterial community structure varies significantly across saliva, supragingival and subgingival biofilm habitats (21, 22), indicating that disease-associated microbial signatures must be interpreted in a niche-specific manner rather than as a single homogeneous oral ecosystem. In addition, epidemiological data indicate that periodontal disease severity is modulated not only by age but also by body weight and breed size, with toy and small breeds exhibiting severe dental crowding that facilitates biofilm retention, anaerobic microenvironment formation, and overgrowth of pathogenic taxa (1, 2).

Rather than being driven by a single pathogen, accumulating ecological and clinical evidence supports a polymicrobial dysbiosis model in which coordinated shifts in multiple taxa collectively drive disease progression (7–9, 16–18). Host inflammatory feedback further exacerbates tissue destruction and promotes persistence of disease-associated microbial communities (23, 24). Although *P. gulae* is frequently detected in canine periodontitis, current evidence does not support its designation as a definitive keystone pathogen. Instead, disease severity appears to emerge from interactions among multiple bacterial taxa and host inflammatory responses. *P. gulae* nevertheless exhibits considerable strain-level heterogeneity: variation in the *fimA* gene encoding its fimbrial adhesins (types A, B, and C) correlates with differences in epithelial adhesion, immune activation, and clinical disease severity (25–27). These findings underscore that canine periodontal pathology is determined not only by community composition but also by genotype-specific virulence traits within dominant taxa.

Recent large-cohort and age-stratified analyses further demonstrate that many microbial biomarkers associated with health and disease are derived from uncharacterized or uncultured taxa, emphasizing a major limitation of sequencing-only approaches (20). While high-throughput surveys robustly define taxonomic associations, they do not resolve the functional contributions of individual species or strains within the biofilm. Therefore, culture-dependent isolation and detailed phenotypic characterization remain essential to determine whether periodontal disease progression is driven by shifts in species abundance, strain-specific virulence, or emergent interactions among previously uncharacterized taxa. This gap between association-based microbiome profiling and mechanistic validation represents a critical barrier to the rational design of precision microbial therapeutics targeting canine oral dysbiosis.

## 3 Microbial therapeutics for oral diseases

### 3.1 Probiotics and postbiotics

Probiotic strategies in canine oral health aim to modulate microbial ecology rather than eliminate bacterial populations. In this context, probiotics and prebiotics are increasingly conceptualized as tools for reshaping dysbiotic oral biofilms through competitive exclusion, metabolic modulation, and host immune regulation rather than through direct bactericidal activity (10, 11). This ecological mode of action is consistent with contemporary dysbiosis-based models of periodontal disease pathogenesis (7, 8). A well-studied example is *Streptococcus salivarius* K12, a human-derived oral probiotic originally isolated from the dorsal surface of the human tongue (28). Although not a native commensal of the canine oral microbiome, *S. salivarius* K12 can transiently colonize the oral cavity in dogs and produce the bacteriocins salivaricin A and B, which inhibit volatile sulfur compound (VSC)-producing anaerobes. In a placebo-controlled clinical trial, Matsumoto et al. (12) demonstrated that daily administration of *S. salivarius* K12 significantly reduced oral malodor and decreased the abundance of halitosis-associated bacteria, supporting functional ecological interference as a therapeutic principle for restoring oral microbial balance in dogs. Notably, the observed effects were maintained only during active administration, underscoring the transient and host-dependent nature of probiotic colonization in the canine oral cavity. Because these evaluations were primarily based on salivary microbiota and halitosis indices, their direct impact on subgingival dysbiosis and periodontal tissue destruction remains uncertain (21, 22).

Composite probiotic formulations have also been investigated. You et al. (13) reported that oral supplementation with *Lactobacillus acidophilus* and *Enterococcus faecium* altered the salivary microbiota composition and significantly reduced gingival inflammation in dogs. These results indicate that canine oral microbial communities are ecologically plastic and responsive to probiotic intervention, yet sustained engraftment of exogenous strains is rarely achieved, and functional benefits are likely mediated through short-term community perturbation rather than long-term species replacement. To date, no study has demonstrated durable integration of probiotic strains into the canine subgingival biofilm ecosystem.

More recently, postbiotics—non-viable microbial cells and their bioactive metabolites—have gained attention due to improved stability, safety, and independence from live colonization. In a canine oral epithelial model, Kim et al. (14) showed that heat-killed *Lactobacillus paracasei* SMB092 upregulated *β*-defensin expression, enhancing mucosal innate defense and reducing the local abundance of *Porphyromonas* and *Fusobacterium*. These findings indicate that postbiotics can exert antimicrobial effects predominantly through host-mediated innate immune activation rather than through direct microbial competition. Translating these findings to clinical application, Sordillo et al. (15) conducted a randomized controlled trial showing that a sustained-delivery postbiotic oral chew derived from lactic-acid-producing bacteria significantly reduced VSC levels and veterinarian-scored halitosis after 4 weeks of administration. For dogs, which cannot voluntarily control swallowing, such chew-based or surface-retentive formulations are particularly important for ensuring prolonged mucosal contact and therapeutic exposure.

Accordingly, technologies that enhance adhesion to the oral mucosa or tooth surface and provide sustained local release—such as chews, gels, or coated food pellets—are likely to be critical determinants of clinical efficacy in canine applications. However, like probiotics, current clinical endpoints have focused primarily on malodor and superficial inflammatory indices rather than on quantitative subgingival microbial shifts or longitudinal periodontal attachment outcomes.

Taken together, probiotics and postbiotics function primarily as ecological stabilizers, enhancing beneficial microbial interactions and mucosal barrier defenses rather than serving as direct antibacterial agents. Their therapeutic effects in dogs are currently supported mainly by short-term clinical and *in vitro* studies, and long-term impacts on periodontal tissue destruction and microbial succession remain largely unexplored. Thus, despite their biological plausibility, probiotics and postbiotics cannot yet substitute for definitive periodontal debridement but may serve as adjunctive strategies within future formulation-optimized microbiome-based management approaches (Table 1; Figure 1).

**Table 1:** Summary of microbial therapeutics investigated for canine periodontal disease.

Therapeutic type	Target organism(s)/pathway	Study model	Observed outcomes	Key limitations
Probiotics (*Streptococcus salivarius* K12; *Lactobacillus acidophilus* MJCD175; multi-strain composite probiotics).	Plaque-associated and volatile sulfur compound (VSC)-producing bacteria; competitive biofilm colonization.	Beagles; client-owned dogs.	Reduced halitosis; modest plaque reduction; shifts in oral microbial community structure (10–13)	Colonization typically short-lived; strain performance varies by host age, oral environment, and baseline microbiome.
Postbiotics (*Lactobacillus paracasei* SMB092; commercially formulated postbiotics).	Induction of β-defensin expression; modulation of local inflammatory tone; reduction of odor-associated taxa.	*In vitro* + randomized controlled canine trial.	Decreased abundance of halitosis-associated bacteria; improved breath odor; evidence of mucosal immune activation (10, 11, 14, 15).	Optimal dosing and duration remain undefined; long-term oral effects not yet evaluated.
Bacteriophage-based treatments.	*Porphyromonas* spp., *Fusobacterium* spp. within multispecies oral biofilms.	Preclinical/translational (*ex vivo* and human oral biofilm models).	Selective suppression of pathogenic taxa; biofilm structural disruption; conceptual fit for targeted dysbiosis control (29–33, 35, 42).	Veterinary oral phage trials not yet conducted; stability, delivery, and regulatory classification remain unresolved.
Predatory bacteria (*Bdellovibrio*, *Micavibrio*).	Gram-negative periodontal pathogens; multispecies biofilms.	*In vitro* human pathogen models; food-industry biofilms.	Reduction of viable Gram-negative pathogens; disruption of biofilm integrity; proof-of-concept for biological predation (40–43).	Evidence currently limited to non-canine *in vitro* models; no *in vivo* canine oral studies to date; biofilm penetration efficiency, host–microbiome safety, and long-term ecological stability in the canine oral niche remain unvalidated.
CRISPR-based/engineered probiotics.	Precision deletion or silencing of virulence factors; targeted suppression without broad community disruption.	Preclinical (human oral model systems).	Genotype- and virulence-specific antimicrobial effects; ability to spare commensal taxa (11, 51, 53, 54).	No companion-animal oral trials to date; safety and persistence of edited strains require regulatory review.
Microbiota transplantation/gut–oral axis modulation.	Systemic immune tone and inflammatory signaling pathways linked to oral disease risk.	Dogs with chronic enteropathies (clinical FMT trials).	Improved immune and inflammatory balance; indirect implications for oral microbial resilience (56, 59, 60).	Not a direct oral microbial therapy; oral colonization effects are inferential and require targeted validation.

### 3.2 Bacteriophage and predatory-bacteria–based therapies

Bacteriophages (phages) selectively infect and lyse bacteria, providing strain-level antimicrobial precision without broadly disrupting commensal microbiota. This narrow host specificity distinguishes phage therapy from conventional antimicrobials and aligns with ecological models of periodontal disease as a dysbiosis-driven disorder rather than a single-pathogen infection (29–31). In human oral models, phages active against *Streptococcus mutans* and *Fusobacterium nucleatum* have been shown to reduce biofilm biomass and viable bacterial counts *in vitro*, while phages targeting *P. gingivalis* have remained largely conceptual (32, 33). Although these studies demonstrate the potential of phages to modulate oral biofilms under controlled conditions, canine oral and periodontal applications remain untested, underscoring the need for veterinary-specific investigations.

Veterinary studies nevertheless provide important translational safety data. Moodley et al. (34) isolated lytic phages targeting *Staphylococcus pseudintermedius* from canine dermatological and soft-tissue infection isolates, demonstrating strain-specific host range and susceptibility to phage infection. Supporting these observations, a recent multi-species synthesis by Bianchessi et al. (35) reported that phage therapy has been well tolerated across both companion and farm animals *via* topical and systemic routes, with minimal adverse effects and variable therapeutic success depending on target species and formulation strategy. Collectively, these studies support the feasibility and biocompatibility of bacteriophages in veterinary medicine; however, controlled oral and periodontal applications in dogs remain largely unexplored.

In canine periodontal disease, *P. gulae* has frequently been selected as a candidate antimicrobial target because its virulence is strongly influenced by *fimA* genotype variation (36). Genomic analyses of *P. gulae* have revealed mobile genetic elements and putative phage-derived sequence fragments, suggesting evolutionary phage–bacterium interactions; however, lytic phages active against *P. gulae* have not yet been isolated, and there is currently no consensus within the veterinary dental community that *P. gulae* represents a key or dominant pathogenic driver of canine periodontitis. Rather, accumulating ecological data support a polymicrobial dysbiosis framework in which multiple taxa jointly contribute to disease progression.

A major translational barrier to both phage therapy and predatory-bacteria–mediated biocontrol is the structural complexity of multispecies subgingival biofilms, in which extracellular polymeric substances impede biological penetration and constrain diffusion (37–39). Experimental studies demonstrate that predatory bacteria such as *Bdellovibrio* and *Micavibrio* can reduce viable Gram-negative human pathogens and disrupt biofilm integrity *in vitro* (40, 41), and translational reviews highlight their potential as antimicrobial alternatives (42, 43). However, all available evidence for predatory bacteria in oral contexts remains confined to human or *in vitro* models.

Additionally, phage-based approaches are consistent with the One Health objective of reducing reliance on broad-spectrum antibiotics and mitigating antimicrobial resistance pressures in both veterinary and human medicine (44). Nevertheless, for canine periodontal disease, phage therapy remains at preclinical stage, with critical gaps in target phage isolation, delivery across dense subgingival biofilms, and long-term ecological safety. Overall, these findings provide a theoretical basis for future biological precision-control strategies in canine periodontitis, but substantial dog-specific experimental validation will be required before clinical translation can be realistically pursued.

### 3.3 Synthetic biology and engineered probiotics

Synthetic biology enables the design of microbial living therapeutics with programmable functions that extend beyond the ecological competition of traditional probiotics. In contrast to conventional supplementation, it emphasizes the rational reprogramming of microbial functions through standardized genetic parts, regulatory circuits, and controlled payload delivery. Current frameworks adopt a modular design strategy encompassing chassis selection, circuit construction, therapeutic payload expression, and biosafety constraints (45, 46).

Formulation science is a key determinant of therapeutic feasibility. Mucoadhesive coatings, encapsulated delivery systems, and surface-retention strategies have been explored to enhance local stability and peptide/protein release (47). However, no engineered probiotic or synthetic-biology–based living therapeutic has yet been validated for oral delivery in dogs. Importantly, the canine oral environment differs substantially from that of humans, with a typically higher oral pH (approximately 7–7.5) and very low salivary *α*-amylase activity, both of which may influence the survival of conventional lactic-acid–based chassis and the functional efficiency of engineered genetic circuits. Adaptation to the canine oral niche will therefore require species-specific optimization of formulation, dosing, and biosafety containment.

CRISPR-based antimicrobial strategies are defined as programmable gene-editing systems that utilize sequence-specific guide RNAs and Cas nucleases to selectively eliminate bacterial strains or disable virulence and resistance genes through targeted DNA cleavage (48–50). Preclinical studies demonstrate sequence-specific elimination of bacterial strains or virulence genes with minimal disruption of commensals (51–53). Given the *fimA* genotype–linked virulence variation of *P. gulae* (36), genotype-selective CRISPR targeting constitutes a plausible theoretical antimicrobial strategy. Nonetheless, delivery into multispecies canine oral biofilms, construct stability in saliva, and prevention of off-target effects or horizontal gene transfer remain unresolved barriers (46).

To address these physiological and ecological constraints, future strategies may benefit from the use of canine-derived oral commensals as alternative chassis candidates, which may exhibit superior niche adaptation, mucosal persistence, and genetic circuit stability compared with human-derived probiotic strains. Overall, synthetic biology and engineered probiotics provide a mechanistically powerful but still preclinical framework for periodontal therapeutics. All supporting data are derived from human or *in vitro* systems, and species-specific chassis selection, genetic circuit optimization for the canine oral environment, and rigorous biosafety validation remain essential for clinical translation (11, 54).

### 3.4 Microbiota transplantation and ecological rebalancing approaches

Microbiota transplantation aims to restore ecological balance by introducing a stable donor-derived microbial consortium. In veterinary medicine, fecal microbiota transplantation (FMT) is increasingly used to manage chronic enteropathies in dogs, with multiple studies demonstrating clinical improvement and partial restoration of microbial diversity (55–57). Case reports further indicate that oral capsule-based FMT is feasible and well tolerated (58) and recent clinical guidelines outline standardized donor screening and administration protocols for companion animals (59). A randomized, double-blinded trial showed that oral FMT can benefit dogs with tylosin-responsive enteropathy, although efficacy varies across dysbiosis phenotypes (60). Controlled safety evaluations in healthy dogs further confirm the biological compatibility of FMT (61).

However, direct transplantation of donor oral microbiota has not yet been validated as a periodontal therapy in dogs. A pilot oral microbiota transplantation (OMT) study in dogs with naturally occurring periodontitis demonstrated only transient transfer of donor-derived microbial signatures without durable community engraftment (62). Because the oral cavity differs fundamentally from the gut in oxygen exposure, continuous salivary flow, and biofilm architecture, FMT principles cannot be directly extrapolated to periodontal applications. Thus, the relevance of microbiota transplantation to periodontal disease remains primarily conceptual: it supports the paradigm that restoration of a health-associated microbial ecosystem, rather than indiscriminate pathogen eradication, can be therapeutically beneficial.

Looking forward, a more rational strategy for canine periodontal disease may involve defined microbial consortia tailored for oral ecological requirements, incorporating strains with stable mucosal adherence, oxygen tolerance, and co-aggregation compatibility. Importantly, this concept can be directly integrated with the synthetic-biology framework discussed in Section 3.3, in which genetic circuit design and chassis engineering could be extended beyond single strains to regulate the stability, functional output, and interspecies interactions of entire microbial consortia. Such engineered consortia may enable controlled community-level functions such as sustained biofilm modulation, spatial niche occupation, and coordinated anti-inflammatory or antimicrobial activities. Because canine periodontal dysbiosis reflects coordinated shifts across multiple interacting taxa, engineered community-level reconstruction rather than whole-microbiota transfer is more likely to provide a feasible and clinically translatable strategy for restoring oral microbial homeostasis.

## 4 Discussion, controversies, and future directions

Although microbial therapeutics offer a promising shift in the management of canine periodontal disease, substantial scientific and translational uncertainties remain. A major limitation is the transient persistence of administered microbial agents in the oral cavity. Most probiotic strains, including *S. salivarius* K12 and various *Lactobacillus* species, exhibit only short-term colonization, with therapeutic effects diminishing after cessation of supplementation (12, 13). Whether durable ecological rebalancing can be achieved through optimized formulations, periodic re-administration, or host-adapted consortia remains unresolved. Moreover, the ecological safety of live microbial agents, including horizontal gene transfer and unintended displacement of native taxa, requires continued evaluation.

Baseline oral microbiota composition varies substantially with age groups, breeds, and health states, complicating the interpretation of intervention outcomes. Most available datasets rely on 16S rRNA amplicon sequencing, which lacks the resolution to discriminate closely related strains or genotype-specific virulence determinants (20). This limitation is particularly evident in *P. gulae*, where *fimA* genotype variation is associated with functionally distinct adhesive and inflammatory phenotypes (26, 27, 36). Accordingly, strain-resolved metagenomics, culturomics, and phenotypic validation are essential to move from association-based profiling toward causal inference.

Clinical evidence for microbial therapeutics also remains limited. Existing trials of probiotics and postbiotics are generally short-term and small in scale, with heterogeneous clinical endpoints (14, 15). Although phage therapy and predatory bacteria show promise in preclinical systems and in non-oral veterinary contexts, no controlled clinical trials have yet evaluated these approaches for canine periodontal disease, and translation is constrained by challenges in biofilm penetration, delivery stability, and long-term ecological safety.

Regulatory and commercialization barriers add further complexity. Microbial therapeutics, including live strains, postbiotics, engineered microbes, and phage-based products, are not yet governed by harmonized veterinary regulatory frameworks, and guidelines for manufacturing, biosafety testing, and environmental containment are still evolving (59), limiting routine clinical implementation.

Importantly, the current lack of scientific consensus regarding a definitive key pathogen driving canine periodontal disease represents a fundamental challenge for the advancement of all targeted therapeutic strategies discussed in this review, including synthetic biology–based approaches, phage therapy, and other precision antimicrobials. Concentrated research efforts aimed at establishing strain-resolved causal relationships within dysbiotic biofilms are therefore critically required.

Despite these challenges, microbial therapeutics continue to offer a compelling framework for future periodontal management. Probiotics and postbiotics demonstrate the feasibility of modulating oral ecological function and reducing indicators such as malodor and inflammation. Advances in synthetic biology, CRISPR-based antimicrobials, phage therapy, and biological predation introduce the possibility of strain- or genotype-level control of virulence rather than broad bacterial suppression. Looking ahead, progress is most likely to emerge from three converging directions: (1) host-adapted, strain-specific probiotics or defined microbial consortia with stable oral retention, (2) targeted biological antimicrobials designed for genotype-specific virulence control, and (3) advanced delivery platforms that prolong residence time within the oral cavity. Together, these approaches support a transition from episodic mechanical intervention toward long-term ecological management of canine periodontal health.
